# Supplementary material for: Exploring the Molecular Origin for the Long-Range Propagation of the Substrate Effect in Unentangled Poly(methyl methacrylate) Films
Source: Polymers (Basel). 2023 Dec 9;15(24):4655. doi: 10.3390/polym15244655 (PMC10748294; doi:10.3390/polym15244655)
Supplement: Supplementary file 1 [file polymers-15-04655-s001.zip › polymers-2759688-supplementary.pdf]

Supplementary Materials for

Exploring the Molecular Origin for the Long-Range Propagation of the Substrate Effect in Unentangled Poly(methyl methacrylate) Films

Jianquan Xu *, Xiaojin Guo, Hongkai Guo, Yizhi Zhang and Xinping Wang *

Institute for School of Chemistry and Chemical Engineering, Key Laboratory of Surface & Interface Science of Polymer Materials of Zhejiang Province, Zhejiang Sci-Tech University, Hangzhou 310018, China

* Correspondence: jqxu@zstu.edu.cn (J.X.); wxinping@zstu.edu.cn (X.W.)

1. Sample preparation and characterization

1.1. The detail of the synthesis process

Linear and ring PMMA labeled by the fluoro-group FMA were synthesized by combining atom transfer radical polymerization (ATRP) [33] and a copper (I)-catalyzed alkyne-azide cycloaddition (CuAAC) “click” reaction [34,48]. Scheme S1 shows the detailed synthetic route and **1-6** is the serial number for the different chemical products from the reaction. The alkyne functionalized initiator, $TMS\text{-}C{\equiv}C\text{-}CH_2OOCC(CH_3)_2Br$ **(2)**, was synthesized as published in the reference [49]. A 50 mL aliquot of THF solution with BIBB (5.6 ml, 50.6 mmol) was added dropwise to a solution of $TMS\text{-}C{\equiv}C\text{-}CH_2OH$ **(1)** (5 ml, 33.7 mmol) and TEA (7ml, 50.6 mmol) in 90 ml THF, while maintaining the reaction temperature at 273 K. After all the solution had dripped off, the reaction solution was stirred for another 5 h at room temperature for the reaction to complete. The triethylammonium salt was removed from the reaction solution by filtration, then the solvent was removed by a rotary evaporator. The crude product was then dissolved in CH_2Cl_2 and washed twice

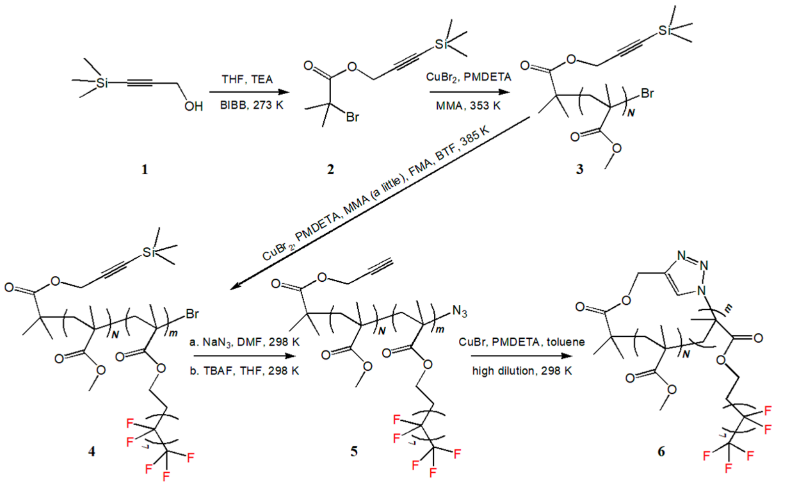

Scheme S1. Synthesis of the fluoro-labeled PMMA with different chain topologies via a combination of ATRP and CuAAC.

with a saturated NH_4Cl solution and twice more with distilled water. The resulting organic layer was dried with anhydrous $MgSO_4$ and subsequently purified by column chromatography (petroleum ether/EtOAc 95:5). The final product resembled a colorless oil. Figure S1 shows the ^{1}H NMR spectrum of product **(2)**, which revealed the alkyne functionalized initiator was successfully synthesized.

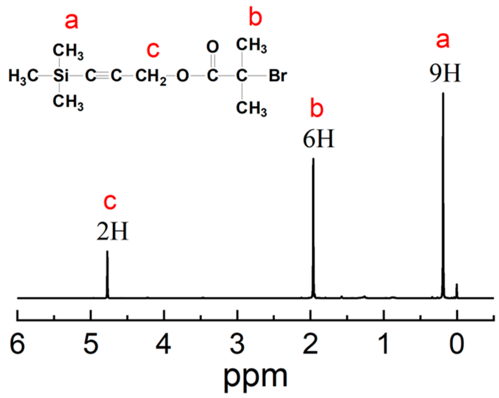

Figure S1. The ^{1}H NMR spectrum of the alkyne functionalized initiator, TMS-C≡C-$CH_2OOCC(CH_3)_2Br$.

The macroinitiator *l*-TMS-C≡C-$PMMA_N$-Br **(3)** and fluoro-tracer-labeled linear PMMA (*l*-TMS-C≡C-$PMMA_N$-*tr*-FMA_m-Br **(4)**) with different molecular weights were synthesized by ATRP similar to our previous work [33]. However, herein, we used Cu(II) as catalyst (which was reduced by MMA to Cu(I)) rather than Cu(I) directly (which is insensitive to moisture and oxygen) to yield lower molecular weights and narrower molecular weight distributions. The number of FMA groups introduced to each chain was 1~4, as detected by ^{19}F NMR using an internal standard method with trifluorotoluene as the internal standard. After obtaining *l*-TMS-C≡C-$PMMA_N$-*tr*-FMA_m-Br **(4)**, an azide reaction was executed [34]. Three grams of *l*-TMS-C≡C-$PMMA_N$-*tr*-FMA_m-Br **(4)** was dissolved in 10 ml of DMF in a

50 ml three necked flask equipped with a magnetic stirrer. NaN_3 (10 equiv.) was added to this solution and the mixture was stirred for 24 h at 298 K. The polymer was then precipitated in cryogenic methanol. Thereafter, 2 g of *l*-TMS-C≡C-PMMA$_N$-*tr*-FMA$_m$-N_3 (5) (i.e., *l*-PMMA$_N$-*tr*-FMA$_m$) was dissolved in 10 ml of THF in a 50 ml three necked flask with 1.5 equiv. of TBAF and stirred for 24 h at 298 K [50]. Then *l*-PMMA$_N$-*tr*-FMA$_m$ (5) was precipitated in cryogenic methanol.

The ring PMMA was synthesized by a copper (I)-catalyzed alkyne-azide cycloaddition (CuAAC) "click" reaction [34,48]. A very dilute solution (0.2 mM) of *l*-PMMA$_N$-*tr*-FMA$_m$ **(5)** in toluene (100 mL) was added in three freeze/pump/thaw cycles to a 250ml flask. In another 500 ml flask, PMDETA (100 equiv.) was dissolved in toluene (200 mL) and degassed in three freeze/pump/thaw cycles, then Cu(I)Br (50 equiv.) was added immediately, followed by two pump-and-thaw cycles. Upon thawing, a syringe with pump was employed to transfer the solution of *l*-PMMA$_N$-*tr*-FMA$_m$ **(5)** to a rapidly stirring solution of Cu(I)Br/PMDETA in toluene at room temperature at a rate of 8 μL/min. After the addition of the polymer solution was completed, the solution was stirred for an additional 2 h. The final product, *r*-PMMA$_N$-*tr*-FMA$_m$ **(6)**, was purified by neutral aluminum oxide to remove the copper salt, followed by precipitation in n-hexane and drying in vacuum.

As reported [34,51], there are two obvious characteristics to verify the cyclization reaction of linear polymer is executed successfully: one is the click reaction is occurred completely, the other is the molecular weight of polymer keeps constant. Figure S2 shows the Fourier Transform Infrared Spectroscopy (FTIR) and Gel

Permeation Chromatography (GPC) spectra of the *l*-$PMMA_{56}$-*tr*-FMA_1 and *r*-$PMMA_{56}$-*tr*-FMA_1 samples. As we can see, the characteristic peak of the azide group at 2100 cm^{-1} in the FTIR spectrum [52] disappeared for the *r*-$PMMA_{56}$-*tr*-FMA_1 sample (Figure S2a), which indicated click reaction is occurred completely. This was double checked by using ^1H NMR (data no shown). Concomitantly, the retention time in the GPC spectrum of the *r*-$PMMA_{56}$-*tr*-FMA_1 became longer than that of the *l*-$PMMA_{56}$-*tr*-FMA_1, which is expected for a ring polymer has a smaller hydrodynamic volume, see Figure S2b. In addition, the inexistence of shoulders in the GPC traces at low retention times in Figure S2b revealed that the content of poisoning impurities, such as linear chains or longer cycles, are low enough in *r*-$PMMA_{56}$-*tr*-FMA_1 sample. Both these findings evidenced that the cyclization reaction occurred successfully with high efficiency. Two different methods were further employed to evaluate the purity of ring polymers, one was the hydrodynamic volume ratio [53-55] and the other was the deconvolutions of GPC spectra [56] between the ring polymer and corresponding linear precursor. For *r*-$PMMA_{56}$-*tr*-FMA_1, the hydrodynamic volume ratio of 0.76 was obtained from Figure S2b, which is in a good agreement with previously reported ones for high purity cyclic polymers and its linear precursor and a level of purity higher than 98 mol %. For other ring samples, the hydrodynamic volume ratio is about 0.76-0.78 and the estimated level of purity is higher than 97 mol %. Similar purities of > 97% were obtained by the deconvolutions of GPC spectra.

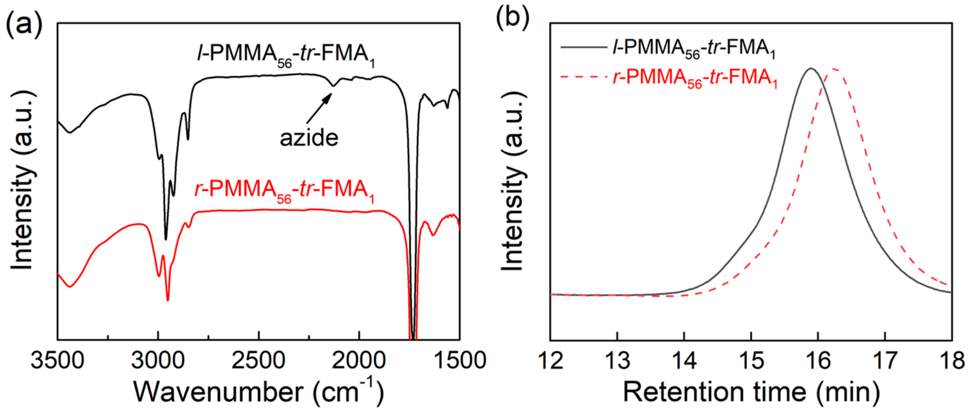

Figure S2. (a) Fourier Transform Infrared Spectroscopy (FTIR) spectra and (b) Gel Permeation Chromatography (GPC) spectra of *l*- and *r*-$PMMA_{56}$-*tr*-FMA_1.

1.2. Sample characterization

The molecular weight distributions of the ring and linear PMMA were determined by GPC (Waters-150C, USA). Solutions of the polymers in THF at a concentration of 1 mg/ml were injected into the GPC at 313 K at a flow rate of 1 mL/min. Table S1 displays the number-average molecular weight, M_n, and polydispersity index (PDI) of the fluoro-labeled polymers used in this work.

Table S1. Characteristics of the *l*-PMMA and *r*-PMMA samples used in the experiments.

Sample	M_n (kg/mol)	PDI
r-$PMMA_{56}$-*tr*-FMA_1	5.6	1.21
r-$PMMA_{129}$-*tr*-FMA_1	12.9	1.20
r-$PMMA_{176}$-*tr*-FMA_1	17.6	1.18
r-$PMMA_{225}$-*tr*-FMA_2	22.5	1.16
l-$PMMA_{56}$-*tr*-FMA_1	5.6	1.19
l-$PMMA_{129}$-*tr*-FMA_1	12.9	1.18
l-$PMMA_{176}$-*tr*-FMA_1	17.6	1.16
l-$PMMA_{225}$-*tr*-FMA_2	22.5	1.17
l-$PMMA_{279}$-*tr*-FMA_2	27.9	1.15
l-$PMMA_{440}$-*tr*-FMA_3	44.0	1.17
l-$PMMA_{672}$-*tr*-FMA_4	67.2	1.18
l-$PMMA_{920}$-*tr*-FMA_4	92.0	1.13

2. References

The references are presented in the main text.
